# Clinical outcomes following Triceps-Reflecting Anconeus pedicle (TRAP) approach for treating intercondylar distal humerus fractures: A systematic review

**DOI:** 10.1007/s00590-025-04617-6

**Published:** 2025-12-08

**Authors:** Shahabeddin Yazdanpanah, Braeden R. Gooch, John W. Cyrus, Benjamin P. Cassidy, Matthew S. Smith, James R. Satalich, Jennifer L. Vanderbeck

**Affiliations:** 1 College of Medicine, Northeast Ohio Medical University, Rootstown, USA; 2 Department of Orthopaedic Surgery, Virginia Commonwealth University, Richmond, USA

**Keywords:** Triceps-reflecting anconeus pedicle, TRAP, Humerus, Distal, Intercondylar, Complication

## Abstract

**Purpose:**

Distal humerus fractures (DHFs) constitute 2% of all fractures, with open reduction and internal fixation (ORIF) often indicated for treatment. Within ORIF literature exists the triceps-reflecting anconeus pedicle (TRAP) approach: an alternative that offers adequate exposure while preserving key soft-tissue structures hypothesised to improve outcomes. Despite this, TRAP-related findings remain poorly collated and lacking consensus. Thus, this systematic review aims to synthesise TRAP-utilising intercondylar DHF ORIF outcomes to guide and inform surgical decision-making.

**Methods:**

A PROSPERO-pre-registered query searched PubMed, Ovid-Embase, Cochrane, and Web of Science on June 26th, 2025, for relevant studies. Demographics, range-of-motion (ROM), and complications were among extracted variables. Frequency-weighted means (FWM) and standard deviations, supplemented by narrative synthesis, constituted data analyses.

**Results:**

Twelve moderate quality observational studies were included (*n* = 295; 59.6% male; FWM age 38.7 ± 10.8 years; follow-up 26.1 ± 9.5 months). All patients underwent TRAP approach DHF ORIF for AO type C fractures (C1 = 29.4%; C2 = 40.7%; C3 = 29.9%), most often from falls or motor vehicle accidents. Union was reported in all cases, and at a FWM 15.7 ± 4.7 weeks. FWM Flexion-extension arc was 113.8˚ ± 12.3, prosupination arc was 157.9˚ ± 8.1, and Mayo Elbow Performance Score was 85.5 ± 8.3. The pooled complication rate was 26.5%, with ulnar nerve-related issues (8.8%) and infection (8.5%) most frequent.

**Conclusion:**

The TRAP approach to DHF ORIF appears to yield favorable union, ROM, and functional outcomes; however, ulnar nerve-related issues and infection warrant relative caution. Future large, randomised trials are needed to better optimise alternative surgical approach selection strategies.

**Level of evidence:**

Level III.

## Introduction

Distal humerus fractures (DHFs) constitute approximately 2% of all fractures and 8% of humerus fractures, with a reported incidence of 5.7–8.3 per 100,000 [1, 2]. Most commonly affecting adults over 50, these injuries harbor an even male: female distribution and typically present as closed extra-articular or intra-articular/intercondylar fractures resultant from a fall [1, 2]. DHF treatment options include nonoperative care, open reduction and internal fixation (ORIF), and arthroplasty [3]. Nonoperative care is generally reserved for non-displaced fractures or for surgically-contraindicated patients [4]. Arthroplasty is commonly employed in elderly patients after nonoperative treatment conclusion or termination in order to avoid stiffening or heterotopic ossification [4, 5]. However, ORIF remains the treatment of choice for a large proportion of other patients, as it allows for stable fracture fixation, early range-of-motion (ROM) mobilisation, and helps to prevent further joint degeneration [4, 6, 7].

A variety of surgical approaches have been described to optimise exposure and elbow access for ORIF in DHFs, commonly categorised based on triceps management as either triceps-off or triceps-on, with the gold standard being the olecranon osteotomy [3, 4, 8–10]. One described alternative is the triceps-reflecting anconeus pedicle (TRAP) approach, first introduced by O’Driscoll in 2000. The TRAP technique is a triceps-off, posterior approach that preserves the innervation of the anconeus muscle and the integrity of the posterolateral ligamentous structures, while tandemly providing robust exposure of the elbow joint’s articular surfaces for ORIF in comparable fashion to other approaches [3, 11, 12].

Despite growing interest, the TRAP approach remains either underrepresented in isolation or pooled amongst other approaches in DHF ORIF literature, limiting risk-benefit understanding [13, 14]. When the TRAP approach is distinguished in the literature, such as in a systematic review by Sharma et al. (2018) on the treatment of intercondylar DHF, the analyses have been underpowered and limited by narrow literature searches which, due to restrictive parameters, do not capture the full breadth of relevant studies [15]. Furthermore, since Sharma et al. (2018), several new studies have been published that tandemly provide further TRAP-related insights. Thus, the purpose of the present systematic review is to comprehensively evaluate clinical outcomes following intercondylar DHF ORIF performed using the TRAP approach alternative, including ROM, functional recovery, and complications. By incorporating a broader and more contemporary body of evidence, this review aims to address the current gap in the literature and inform surgical approach-planning regarding the viability of TRAP.

## Methods

### Initial search process and study creation

Available published literature examining ROM, functional recovery, and complications following the TRAP approach to intercondylar humerus fractures were systematically reviewed utilising PubMed, Ovid-Embase, Cochrane, and Web of Science from database inception until June 26th, 2025. A grey literature search was also performed on June 26th, 2025, examining the first five pages of Google Scholar. Search terms used in PubMed were (“triceps-reflecting anconeus pedicle” OR “triceps reflecting anconeus pedicle” OR TRAP) AND (humerus[tiab] OR humeral[tiab] OR “Humerus“[Mesh] OR “Humeral Fractures“[Mesh]). Ovid-Embase search terms were (“triceps-reflecting anconeus pedicle” or “triceps reflecting anconeus pedicle” or TRAP).mp., combined with ((humerus or humeral).ti, ab, kf. or exp humerus fracture/ or exp humerus/). Search terms used in Cochrane were (“triceps-reflecting anconeus pedicle” OR “triceps reflecting anconeus pedicle” OR TRAP) AND (humerus OR humeral OR [mh “Humerus”] OR [mh “Humeral Fractures”]). Web of Science search terms were TS=((“triceps-reflecting anconeus pedicle” OR “triceps reflecting anconeus pedicle” OR TRAP) AND (humerus OR humeral)). Grey literature search terms were (“triceps-reflecting anconeus pedicle” OR “triceps reflecting anconeus pedicle” OR “TRAP”) AND (“humerus” OR humeral”). The structure of this study followed the guidelines of the most recent Preferred Reporting Items for Systematic Reviews and Meta-Analyses (PRISMA) [16]. Additionally, this study was pre-registered in the PROSPERO registry for systematic reviews and meta-analyses (CRD420251075208).

### Inclusion and exclusion criteria

Studies that investigated the outcomes and/or management of intercondylar humerus fractures in adult patients (≥ 18 years) via the TRAP approach to ORIF fracture treatment met inclusion criteria. Data or details on ROM, functional recovery, and/or complications after surgery must have been reported. Exclusion criteria were non-human (cadaveric) studies, non-TRAP studies, non-intercondylar/intra-articular humerus fracture studies, studies containing any pediatric data, non-operative-only studies, case reports, surveys, letters to the editor, reviews, and meta-analyses. Additional exclusion criteria were studies without data, studies without published full-texts, and non-English studies.

### Article screening process

Once the search algorithm was applied to the four databases for the initial query, all articles were uploaded into Rayyan, a public website used for systematic reviews [17]. An independent screener both manually de-duplicated and screened the query abstract results initially based on title and abstract contents (S.Y.). These results were then validated by a second independent screener (B.G.), with any disagreements resolved by the first author. The screening process was immediately followed by full-text screening based on inclusion and exclusion criteria.

### Extraction process

Two independent authors completed data extraction, which included first author, year of publication, number of patients, sex, age, follow-up, fracture-related details, ROM outcomes, functional recovery results, complications, and any other relevant qualitative and quantitative data for statistical analysis and narrative reporting (S.Y. & B.G.). Any conflicts that arose during the data extraction process were resolved by the first author.

## Article quality grading

The studies included in this systematic review were classified as either “non-comparative” or “comparative” for appropriate quality grading using the Methodological Index for Non-Randomized Studies (MINORS) scale [18]. Non-comparative studies were graded out of 16 points and comparative studies were graded out of 24 points. For non-comparative studies, there are 8 items on the scale, and for comparative studies, there are 12 items on the scale, with each item being given either 0, 1, or 2 points. Based on accumulated points and non-comparative/comparative nature, studies were graded as “high quality”, “moderate quality”, or “low quality”. For non-comparative studies, high quality articles scored 16 points, moderate quality articles scored 10–15 points, and low quality articles scored less than 10 points [19]. For comparative studies, high quality articles scored 24 points, moderate quality articles scored 15–23 points, and low quality articles scored less than 15 points [19].

### Statistical analysis

Microsoft Excel (Redmond, WA: Microsoft Corporation) was utilised for this study’s statistical analyses. Frequency-weighted means (FWMs) and other descriptive statistics such as percentages and ranges were used to describe data due to reporting heterogeneity. In instances where temporal heterogeneity was present, such as fracture time-to-union reported in months instead of weeks, all value reporting types were converted to the value reporting type that followed the majority trend, such as months being converted to weeks in the aforementioned example. Furthermore, for estimating the conversion of ranges to standard deviations (SDs) in cases of heterogenic data synthesis, an extrapolated approach to the standards described by Hozo et al. [20] was adopted. Specific to flexion-extension arc calculations, if an arc was not reported, individual extension values were subtracted from flexion values following the elucidation of this method in prior literature [21]. Prosupination arc calculations were performed in a similar fashion, as pronation and supination values were added together, tandemly following literature standards [21].

## Results

### Study selection results

**Fig. 1:**
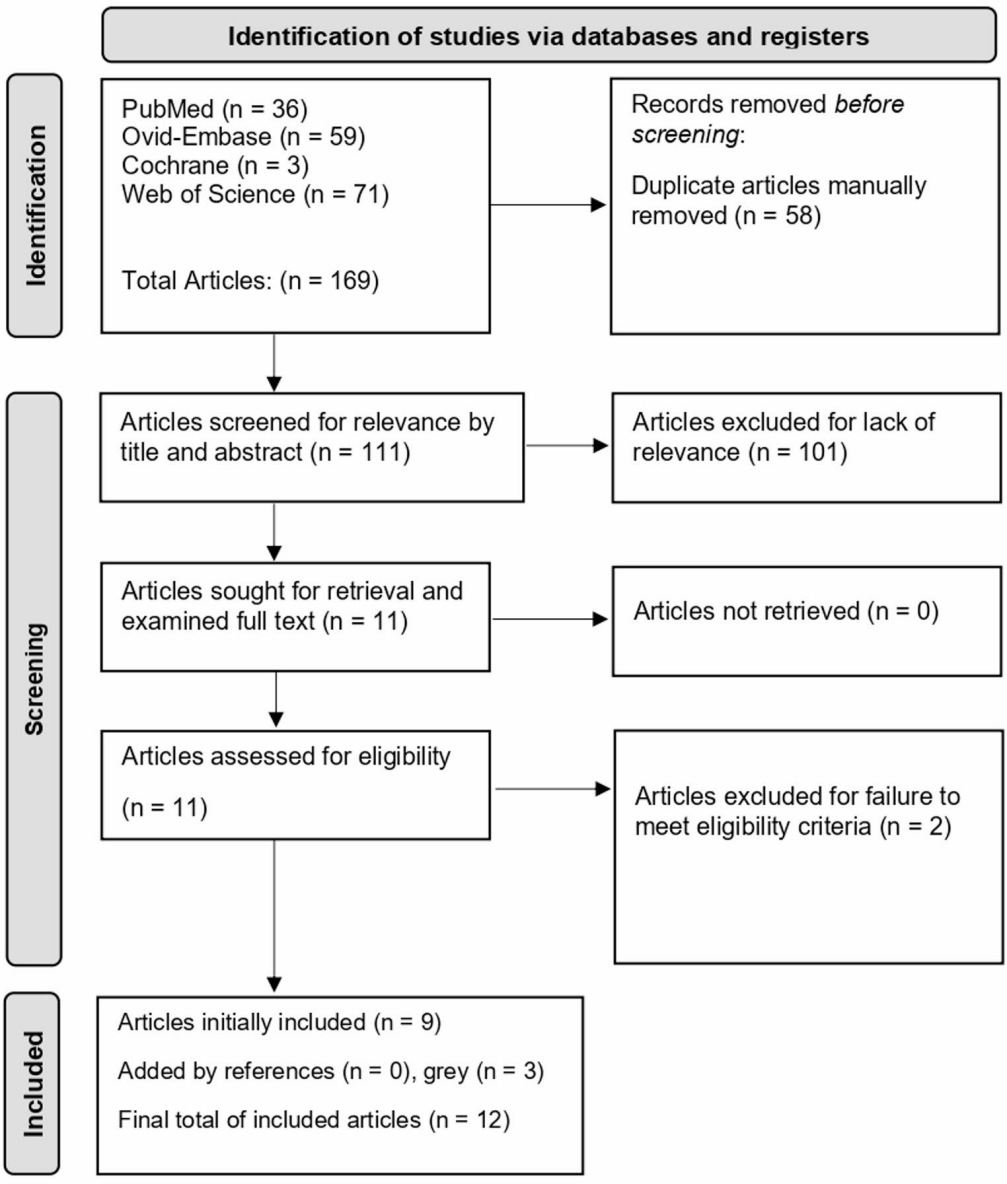
The Preferred Reporting Items for Systematic Reviews and Meta-Analyses (PRISMA) diagram followed in the present systematic review

A total of 169 articles were identified by the search algorithm; after manual de-duplication, 111 remained. Title and abstract screening led to 11 articles sought for retrieval for full-text analysis. Two of those failed to meet eligibility criteria, leaving nine for inclusion. The grey literature search yielded three more articles. After this process, twelve total articles met inclusion criteria and progressed to data extraction (Fig. 1) [22–33].

### Article quality and certainty results

Of the twelve studies extracted, nine were retrospective and three were prospective. There were six non-comparative and six comparative studies. The mean MINORS score was 15.3 ± 4.4 for all studies combined. Mean MINORS score for non-comparative studies (*n* = 6) was 11.3 ± 0.8 (out of 16.0 points). Mean MINORS score for comparative studies (*n* = 6) was 19.2 ± 2.0 (out of 24.0 points). Ultimately, all twelve studies were determined to be of moderate quality (Table 1).

**Table 1:** The methodological index for Non-Randomized studies (MINORS) grading for all included studies.

Author (Year)	Total MINORS Score	Clearly stated aim	Inclusion of consecutive patients	Prospective collection of data	End points appropriate to study aim	Unbiased assessment of study end point	Follow-up period appropriate to study aim	Less than 5% lost to follow-up	Prospective calculation of the study size	Adequate control group	Contemporary groups	Baseline equivalence of groups	Adequate statistical analysis
Ailani (2024)	20	2	2	2	2	1	2	1	0	2	2	2	2
Azboy (2016)	18	2	1	0	2	1	2	2	0	2	2	2	2
Chou (2016)	11	2	2	0	2	1	2	2	0	X	X	X	X
Jain (2023)	18	2	2	1	2	1	2	1	0	2	2	2	1
Jitrapaikulsarn (2023)	11	2	2	0	2	1	2	2	0	X	X	X	X
Mishra (2010)	13	2	2	2	2	1	2	2	0	X	X	X	X
Mittal (2021)	23	2	2	2	2	1	2	2	2	2	2	2	2
Nayak (2023)	18	2	2	0	2	1	2	1	0	2	2	2	2
Ozer (2005)	11	2	2	0	2	1	2	2	0	X	X	X	X
Pankaj (2007)	11	2	2	0	2	1	2	2	0	X	X	X	X
Sachdev (2017)	18	2	1	0	2	1	2	2	0	2	2	2	2
Verma (2019)	11	2	2	0	2	1	2	2	0	X	X	X	X

An “X” was used to occupy a category cell reserved for comparative studies when evaluating a non-comparative study

### Patient and study characteristics

Data from 295 patients undergoing TRAP fixation for intercondylar DHFs were pooled and yielded a FWM age of 38.7 ± 10.8 years, follow-up of 26.1 ± 9.5 months (66.7% reporting), male sex of 59.6%, with the right humerus affected in 54.2% of the cohort (75% reporting) (Table 2). The most common mechanisms of injury were falls, followed then by motor vehicle accidents and bicycle accidents. All studies reported only type-C fractures, following the Arbeitsgemeinschaft für Osteosynthesefragen/Association of the Study of Internal Fixation (AO/ASIF) fracture classification system elucidated in Müller et al. (1990) [34]. C1 fractures comprised 29.4% of the study cohort, while C2 and C3 fractures comprised 40.7% and 29.9%, respectively. The vast majority of these AO/ASIF classifications were closed fractures, followed then by Gustilo-Anderson classified Type 1 open fractures, with many studies excluding Type 2 and 3 open fractures (Table 2) [35].

**Table 2:** Demographics, study, and fracture-related details

Author (year)	Sample size	Average age in years (SD/Range)	Average follow-up in months (SD/Range)	Male %	Laterality % right	Mechanism of Injury	AO/ASIF classification	Closed/Open fracture details
Ailani (2024)	20	43.2 (18–58)	≥ 12	40%	N.R.	Fall: 16 MVA: 4	C1: 7 C2: 11 C3: 3	Closed Type 1 Open
Azboy (2016)	22	37.8 (17–70)	36 (16–74)	60%	N.R.	Fall: 16 MVA: 3 Sports: 2 Firearm: 1	C1: 4 C2: 8 C3: 10	Closed: 18 Type 1: 2 Type 2: 2 Type 3: 0
Chou (2016)	48	41 (18–74)	44.2 (24–88)	58.7%	60.4%	Fall: 11 MVA: 28 Bicycle Accident: 9	C1: 0 C2: 21 C3: 27	N.R.
Jain (2023)	10	43 (N.R.)	≥ 12	40%	45%	Undistinguishable	C1: 14 C2: 4 C3: 2	Closed Type 1 Open
Jitrapaikulsarn (2023)	22	44.3 (21–63)	18.6 (12–32)	40.9%	21.7%	Fall: 3 MVA: 17 Sports: 1 Bicycle Accident: 1	C1: 3 C2: 17 C3: 2	Closed: 17 Unspecified Open: 5
Mishra (2010)	15	31.9 (16–57)	9 (6–14)	86.7%	53.3%	Fall: 12 MVA: 3	C1: 1 C2: 3 C3: 11	Closed: 14 Unspecified Open: 1
Mittal (2021)	32	35.2 (13.1)	23.3 (4.9)	70.5%	43.1%	Fall: 21 MVA: 6 Assault: 1 Other: 4	C1: 7 C2: 14 C3: 11	Closed: 27 Type 1: 5
Nayak (2023)	17	42.5 (N.R.)	≥ 12	41.2%	52.9%	Falls most prevalent	C1: 5 C2: 9 C3: 3	Closed Type 1 Open
Ozer (2005)	11	58.3 (16–70)	26 (14–40)	54.5%	36.4%	Fall: 9 MVA: 2	C1: 4 C2: 4 C3: 2	Closed: 8 Type 1: 3
Pankaj (2007)	40	32 (4.5)	18 (4)	70%	67.5%	Fall: 20 MVA: 16 Direct Trauma: 4	C1: 17 C2: 15 C3: 8	Closed: 36 Type 1: 4
Sachdev (2017)	28	41 (21–65)	≥ 12	57.1%	N.R.	Cases included falls and MVAs	C1: 5 C2: 11 C3: 12	Closed
Verma (2019)	30	32 (4.5)	18 (4)	70%	73.3%	Fall: 15 MVA: 12 Direct Trauma: 3	C1: 16 C2: 10 C3: 4	Closed: 24 Type 1: 6

MVA, motor vehicle accident; N.R., not reported; U.D., Undistinguishable, TRAP data pooled with non-TRAP data

### Operative details and outcomes

The FWM time-to-surgery was 5.1 ± 3.3 days (58.3% reporting), and fracture time-to-union was 15.7 ± 4.7 weeks (58.3% reporting) (Table 3). All studies explicitly reported that either fracture union was observed in all patients, that there were zero cases of nonunion, or that there were no union-related complications. Qualitatively, six studies reported rehabilitative mobilisation within three days postoperatively, while the other six mentioned rehabilitative mobilisation occurring within two weeks. Nine studies mentioned prohibiting or limiting active exercises such as extension and weight-bearing, with the vast majority of this group setting a six-week target. The FWM elbow flexion-extension arc was 113.8˚ ± 12.3, and prosupination arc was 157.9˚ ± 8.1 (41.7% reporting). In most studies where pronation/supination, or prosupination arc were not reported, it was noted that no patients had any pronation/supination limitations. Nine studies reported Mayo Elbow Performance Scores (MEPS), of which the FWM at final follow-up was 85.5 ± 8.3 (75% reporting). Following standard MEPS categorisation, 45.5% of the pooled cohort were classified “excellent”, followed by 39.3% “good”, 11.8% “fair”, and only 3.3% “poor” (Table 3) [36].

**Table 3:** Operative outcomes and details.

Author (Year)	Average time-to-surgery in days (SD/Range)	Average fracture time-to-union in weeks (SD/Range)	Postoperative rehabilitation details	Average elbow flexion-extension arc of motion in degrees (SD/Range)	Average forearm pronation/supination or prosupination arc in degrees (SD/Range) or details	Average MEPS at final follow-up (SD/Range)	MEPS grading at final follow-up
Ailani (2024)	N.R.	13.1 (10–16)	Mobilisation started after 1 week	107 (3.5)	N.R.	84.2 (2.9)	Excellent: 14 Good: 3 Fair: 2 Poor: 1
Azboy (2016)	3.8 (1–12)	N.R.	Mobilisation started after 2 days, active extension prohibited until 6 weeks	108 (70–140)	No patients had pronation/supination limitations	85.9 (55–100)	Excellent: 10 Good: 8 Fair: 1 Poor: 0
Chou (2016)	3.6 (0–9)	< 26	Mobilisation started after 2 days, active exercises prohibited until 6 weeks	121 (100–140)	163 (150–180)	81 (75–100)	Excellent: 14 Good: 26 Fair: 6 Poor: 2
Jain (2023)	5.6 (N.R.)	13.1 (N.R.)	Mobilisation started after 2 days, active exercises prohibited until 6–8 weeks	Flexion: 118 (7.3) Extension: 11 (3.8)	Pronation: 80.8 (N.R.) Supination: 72.3 (N.R.)	84.3 (N.R.)	Excellent: 4 Good: 4 Fair: 1 Poor: 1
Jitrapaikulsarn (2023)	0.8 (0.3–2.5)	16.5 (12–22)	Mobilisation started after 2 weeks, active exercises prohibited until 6 weeks	118.2 (90–135)	N.R.	91.6 (75–100)	Excellent: 13 Good: 9 Fair: 0 Poor: 0
Mishra (2010)	7 (3–26)	< 26 weeks	Mobilisation started after 3 days, active exercises limited until 6 weeks	101.3 (17.1)	Pronation: 90 (0) Supination: 90 (0)	85 (60–100)	Excellent: 6 Good: 4 Fair: 5 Poor: 0
Mittal (2021)	9.9 (4.3)	23.1 (0.5)	Mobilisation started after 3 days, no mention of exercise prohibition	114.5 (14.6)	Pronation: 74.1 (8.0) Supination: 70.3 (6.9)	85.5 (8.2)	Excellent: 14 Good: 16 Fair: 2 Poor: 0
Nayak (2023)	5.8 (N.R.)	12.8 (N.R.)	Mobilisation started after 2 weeks, active exercises limited until 6–8 weeks	Flexion: 117.5 Extension: 11.8	Pronation: 80.1 (N.R.) Supination: 71.8 (N.R.)	85.1 (N.R.)	Excellent: 7 Good: 7 Fair: 2 Poor: 1
Ozer (2005)	N.R.	10–14	Mobilisation started after 2 weeks, no mention of exercise prohibition	110.5 (18.8)	No patients had pronation/supination limitations	N.R.	N.R.
Pankaj (2007)	< 7 days	13.9 (6.5)	Mobilisation started after 2 weeks, active exercises prohibited until 8 weeks	118.4 (80–130)	No patients had pronation/supination limitations	N.R.	N.R.
Sachdev (2017)	N.R.	N.R.	Mobilisation started after 2 days, active exercises prohibited for an unspecified time	110 (85–135)	N.R.	89.9 (55–100)	Excellent: 14 Good: 6 Fair: 6 Poor: 2
Verma (2019)	< 7 days	13.9 (6.5)	Mobilisation started after 2 weeks, active exercises prohibited until 8 weeks	118.4 (7)	No patients had pronation/supination limitations	N.R.	N.R.

SD, standard deviation; MEPS, Mayo elbow performance Score; N.R., not reported

### Complications

All authors reported complications in this cohort of studies, and a FWM complication rate of 26.5% was determined. The most frequently reported complications were ulnar nerve-related issues at a rate of 32.1% of all complications and an 8.8% overall cohort rate, followed by infection at 27.2% and an 8.5% overall cohort rate, triceps weakness at 13.6% and a 3.7% overall cohort rate, and hardware-related issues at 11.1% and a 3.1% overall cohort rate (Table 4). Elbow deformity and skin necrosis were the two least commonly reported complications, each occurring at a 2.5% rate in the complication pool and a 0.7% overall cohort rate. Notably, there was considerable heterogeneity in how authors reported complications; 50% of authors listed instances of heterotopic ossification and hardware-related issues separately from their defined complications. These details are further clarified in Table 4.

**Table 4:** Complications and associated details for included studies.

Author (Year)	Complication %	Author-reported complication details (*N*)	Notable complication exclusions by authors
Ailani (2024)	25%	Infection (3), ulnar nerve-related issues (2), hardware-related issues (2)	N.R.
Azboy (2016)	27.2%	Infection (1), ulnar nerve-related issues (2), triceps weakness (2), elbow deformity (1)	N.R.
Chou (2016)	33.3%	Infection (2), ulnar nerve-related issues (4), triceps weakness (4), soreness (6)	Radiographic findings such as heterotopic ossification, union gaps, and hardware removal were not considered complications
Jain (2023)	40%	Infection (2), ulnar nerve-related issues (1), triceps weakness (1)	Hardware prominence was not considered a complication
Jitrapaikulsarn (2023)	18.2%	Ulnar nerve-related issues (3), heterotopic ossification/stiffness (1)	Heterotopic ossification/stiffness was not considered a complication
Mishra (2010)	40%	Infection (3), ulnar nerve-related issues (1), hardware-related issues (1), heterotopic ossification/stiffness (1)	N.R.
Mittal (2021)	40.1%	Infection (3), ulnar nerve-related issues (2), hardware-related issues (6), skin necrosis (2)	N.R.
Nayak (2023)	38%	Infection (4), ulnar nerve-related issues (2), triceps weakness (2)	N.R.
Ozer (2005)	27.3%	Ulnar nerve-related issues (2), heterotopic ossification/stiffness (1)	N.R.
Pankaj (2007)	7.5%	Infection (1), ulnar nerve-related issues (2)	Implant removal was not considered a complication
Sachdev (2017)	32.1%	Infection (2), ulnar nerve-related issues (3), triceps weakness (2), elbow deformity (1)	Implant impingement was not considered a complication
Verma (2019)	10%	Infection (1), ulnar nerve-related issues (2)	Implant removal was not considered a complication

N, number; N.R., not reported

## DISCUSSION

This study evaluated clinical outcomes following DHF ORIF via TRAP to assess approach viability. Across included studies, fracture union was consistently achieved, with acceptable postoperative ROM and functional outcome scores. The cohort’s pooled complication rate was 26.5%, composed primarily of ulnar nerve-related complications, followed then by infection, triceps weakness, and hardware-related issues. As the first systematic review to isolate such TRAP outcomes, this study offers a novel, collated overview of the clinical trajectory that may be generally anticipated by surgeons considering the TRAP alternative approach to DHF ORIF management. Though, interpretation should be tempered given the overall quality of the studies included.

The union findings within this review serve as a potential marker of the efficacy of the TRAP approach in achieving its primary objective; and this outcome aligns very closely with what is reported in the literature of other operative approaches to intercondylar DHF ORIF. For example, systematic reviews examining olecranon osteotomies report original fracture nonunion rates ranging from 1% to 5% [14, 37]. Furthermore, nonunion of the olecranon osteotomy itself has been reported at rates ranging from < 1% to 5.4%: a modest, yet well-recognised additional complication of this approach that is readily avoided in soft tissue alternatives like TRAP [14, 37–39]. In reviews focused on the paratricipital approach, nonunion of the original fracture has similarly been reported at 0%, while pooled data across multiple intercondylar DHF ORIF approaches have yielded an overall nonunion rate of 3.4% [37, 40]. These comparisons suggest that the TRAP approach may be a comparable alternative to various other approaches with respect to union outcomes. Moreover, while time-to-union details are often underreported in large-scale reviews, the various approach averages reported in individual studies (with multi-year follow-up windows) have included 12 weeks for paratricipital, 14 weeks for olecranon osteotomy, and 16 weeks for posterior transolecranon, providing valuable context for the 15.7 week FWM time-to-union observed in the present review [41–43]. Such findings cautiously support the TRAP approach as a viable surgical alternative in terms of additional union metrics, offering potentially realistic benchmarks for use in clinical shared decision-making.

ROM outcomes demonstrated similar findings. Flexion-extension arcs reported in systematic reviews of intercondylar DHF ORIF, which include mixed approaches such as olecranon osteotomies and triceps sparing techniques, range from 90˚ to 118˚: a spectrum that encompasses the present review’s FWM of 113.8˚ [13]. This is further corroborated by Stoddart et al. (2024), who reported a pooled flexion-extension arc of 104.1˚ in their systematic review of mixed intercondylar DHF ORIF approaches [40]. The prosupination arc means of 160.6˚ for medial-lateral approaches and 154.1˚ for olecranon osteotomies also bracket the FWM prosupination arc of 157.9˚ determined in the present review [44]. Moreover, as all other included studies reported no limitations in prosupination arc in lieu of providing granular data, the TRAP approach is further supported as a technique that preserves postoperative elbow rotation. Morrey et al. (2005) described functional elbow ROM as 100˚ of flexion-extension arc and 100˚ of prosupination arc [45]. Thus, the TRAP approach appears to restore functionally sufficient elbow motion following union, which may be a valuable finding for cautious conveying during patient counseling.

Functional outcomes followed similar trends as those mentioned above, with the FWM MEPS of 85.5 corresponding to an average classification of “good” and bordering “excellent” per established scoring criteria [46]. In a systematic review by Chen et al. (2017), the proportion of elbows rated as “excellent” or “good” ranged from 64.8% to 89.3% in olecranon osteotomy studies and from 70.7% to 100% in triceps sparing studies for intercondylar DHF ORIF, which is highly comparable to the 84.8% combined “excellent” or “good” rating observed in the present review [13]. This is further substantiated by Stoddart et al. (2024), who reported a mean MEPS of 85.0, and Sharma et al. (2018), who reported mean MEPS ranging from 76 for the Campbell approach to 94.9 for the paratricipital approach [15, 40]. Collectively, these comparisons suggest that TRAP is very similar to other approaches with respect to elbow performance metrics, adding support to its candidacy as a viable option for handling intercondylar DHFs.

When discussing complications, however, the TRAP approach presents some concerns. Although the pooled FWM complication rate of 26.5% is substantially lower than the 42.4% rate reported in the systematic review by Stoddart et al. (2024), it closely mirrors the 26.3% rate in a separate systematic review of intercondylar DHF ORIF olecranon osteotomies [40, 47]. The TRAP approach was initially theorised to result in fewer complications compared to the olecranon osteotomy, particularly given its preservation of elbow anatomy; however, the pooled complication data from this review posit challenging to that assumption on a larger scale. There have been several comparative studies that suggest higher complication rates in olecranon osteotomy cohorts relative to TRAP, as well as others that report the opposite, resulting in no clear consensus; this is particularly contentious given that such individual studies are underpowered when compared to both the present systematic review and the broader body of literature on olecranon osteotomies [22, 23, 25, 28, 29, 32]. The rationale for increased complications in olecranon osteotomies includes factors such as nonunion or delayed union at the osteotomy site in addition to the original fracture, hardware-related complications, and symptomatic osteotomy fixation [22, 48]. Nonetheless, these suppositions remain to be validated through higher level-of-evidence comparative studies, and the findings from the present study cautiously suggest that the complication rates between these two approaches may be more comparable than previously hypothesised.

While complications as a broad conglomerate remain a contentious comparative subject, specific complications appear more directly comparable. Beginning with ulnar nerve-related issues, the present study determined an 8.8% cohort rate, which falls within the range of complication rates reported in an intercondylar DHF ORIF systematic review by Jeong et al. (2022), specifically from 3.9% for the paratricipital approach to 12.5% for olecranon osteotomies [37]. However, the 32.1% complication proportion of ulnar nerve-related issues is notably higher than the 8% reported in the systematic review by Yetter et al. (2021), which pooled data from 83 studies evaluating various intercondylar DHF ORIF surgical approaches [14]. This discrepancy may indicate potential cohort-level data skewing, bias in either set of included studies, or underreporting of preoperative neuropathies that are then discovered postoperatively, or it may suggest that the TRAP approach lies on the higher end of ulnar nerve-related complication rates [39]. It should be known that the ulnar nerve was handled in some manner in all included studies, routinely via subcutaneous transposition during operative exposure, as is often standard in TRAP and other DHF ORIF approaches [23, 28]. Furthermore, ulnar nerve transposition itself has been shown to be associated with increased rates of ulnar nerve-related complications [49]. Therefore, the notion that such symptoms persist in TRAP cohorts is not unexpected; however, the higher rates may reflect the technical demands of this approach and its potential to place greater intraoperative or postoperative stress on the ulnar nerve. Regardless of cause, however, this finding is an important clinical distinction for surgeons who are considering the TRAP approach for intercondylar DHF ORIF.

Infection followed ulnar nerve-related issues as the second most frequent complication (cohort rate = 8.5%; complication proportion = 27.2%). Jeong et al. (2022) reported an infection rate ranging from 0.8% in the paratricipital approach cohort to 7.3% in the olecranon osteotomy cohort, denoting both as either marginally or substantially lower than what was observed in the present review [37]. This can be further explored via side-by-side comparisons with a 7% infection rate reported amongst olecranon osteotomy studies in Feinstein et al. (2023), as well as a 4.2% complication proportion observed in the systematic review of olecranon osteotomies by Spierings et al. (2022) [38, 47]. When broadening the scope further to all DHF ORIF approaches, Stoddart et al. (2024) reported an overall infection rate of 6.9%, again falling below what was observed in the current analysis [40]. While infections were not as numerically frequent as ulnar nerve-related complications in this cohort of studies, they arguably pose a greater risk to patient safety when the margins are narratively compared to those of alternative approaches. This can be plausibly explained by the triceps-reflecting nature of the TRAP approach, increasing soft tissue trauma and vascular compromise, which are known infection-related risk factors in the elbow [50]. Infections at the triceps detachment site have also been specifically documented in association with the TRAP approach, and as olecranon osteotomies have been well-described to afford the widest surgical exposure, such benefit may allow for more effective irrigation and hematoma clearance, for example, offering a relative protective effect [27, 51]. Given such hypotheses, infection must remain a mainstay of perioperative counseling, particularly as its comparative risk may be elevated in patients undergoing DHF ORIF via the TRAP approach as an alternative.

Lastly, triceps weakness and hardware-related issues followed in complication frequency, with modest cohort rates of 3.7% and 3.1% and complication proportions of 13.6% and 11.1%, respectively. Triceps weakness has been reported to represent only 0.1% of complications in systematic reviews of olecranon osteotomy intercondylar DHF ORIF, which may directly reflect the greater triceps involvement inherent to the TRAP approach [14]. Within the literature, such TRAP-specific triceps-related complications have been attributed to factors like extensive dissection, improper length-tensioning during reattachment, and rehabilitation protocols that inadequately balance elbow strengthening with soft tissue recovery and maintenance [24, 27]. In contrast, implant removal alone has been documented to account for 9.2% of complications in similar olecranon osteotomy systematic reviews, with an additional 5% representing other hardware-related issues [14]. These findings and comparisons more closely align with the hardware-related complications typically associated with olecranon osteotomies, as previously discussed, and suggest that patients undergoing the TRAP approach may expect a lower relative hardware-related burden [22, 48].

Although this study positions the TRAP approach to intercondylar DHF ORIF as a viable alternative, its findings must be considered within the context of several limitations. First, this systematic review is primarily composed of retrospective, moderate quality observational studies, as assessed by MINORS, which inherently carry risks of biases and limit the strength of syntheses. Though, this also reflects the relative paucity of literature on TRAP, which remains incompletely understood compared to other intercondylar DHF ORIF approaches. As a result, many of the included studies had small sample sizes, comparatively reducing the study cohort size: an important factor to consider when evaluating literature parallels made. Heterogeneity in data reporting was also observed, especially regarding ROM and SD estimates: the latter of which were reported directly from the original studies, with constraints on more advanced statistical manipulations attributed to study designs. The estimates that did occur, however, were calculated using peer-reviewed, widely accepted methodologies. Other limitations included low reporting rates, missing data for certain variables, and the possibility of reporting bias given the uniformly observed union outcomes. Such considerations hindered advanced analyses and warrant further caution considering overall study qualities, designs, and sample sizes. Of note, although all included studies reported AO/ASIF classification type C DHFs, variability in fracture severity was present: some studies included only closed fractures or type 1 open fractures, whereas others also incorporated type 2. While recent literature suggests no significant differences in outcomes between closed and open type C DHFs, the inclusion of more severe injuries, even if limited and transparently described, should be acknowledged [52]. Finally, the pooled complication rate relied on author-reported data, with variability in how specific complications were classified. For example, heterotopic ossification was inconsistently categorised across studies. Such heterogeneity in author-defined complications is a well-recognised source of bias in surgical literature, and although this was addressed transparently in the present study, it must tandemly be considered during interpretation [53].

Despite these limitations, to the authors’ best knowledge, the present systematic review still represents the first, most comprehensive, and largest analysis of clinical outcomes following the TRAP approach to intercondylar DHF ORIF. As such, it serves as a valuable reference point and foundation for clinical decision-making and contemporary research. To further validate and expand upon these findings, future high quality, large randomised controlled trials are warranted to more appropriately compare TRAP to other surgical approaches for intercondylar DHF ORIF, with careful efforts to account for aforementioned limitations.

## Conclusion

In this novel systematic review of the TRAP approach for intercondylar DHF ORIF, all reported patient cases achieved fracture union, and postoperative ROM and MEPS were comparable to appropriately relevant literature. Complications, however, highlighted some concerns: ulnar nerve-related issues and infections were the two most frequent, with rates often exceeding literature comparisons. This was presumed to reflect the TRAP approach’s anatomic and technical nuances. Likely for similar reasons, hardware-related complications were rare. Thus, TRAP may generate acceptable functional outcomes and a relatively low hardware burden, though can be accompanied by a notable complication profile. Accordingly, the findings presented within this review merit careful interpretation in recognition of inherent limitations and potential biases. For corroboration, future large, high quality randomised controlled trials are warranted to further clarify inter-approach differences, improve data homogeneity, and standardise outcome reporting. Until then, surgeons should cautiously weigh the potential risks and benefits of TRAP when considering the most appropriate, practical, and measured approach for intercondylar DHF ORIF.

## Data Availability

No datasets were generated or analysed during the current study.
